# 3D-Bioprinted Co-Cultures of Glioblastoma Multiforme and Mesenchymal Stromal Cells Indicate a Role for Perivascular Niche Cells in Shaping Glioma Chemokine Microenvironment

**DOI:** 10.3390/cells13171404

**Published:** 2024-08-23

**Authors:** Katarzyna Zielniok, Kinga Rusinek, Anna Słysz, Mieszko Lachota, Ewa Bączyńska, Natalia Wiewiórska-Krata, Anna Szpakowska, Martyna Ciepielak, Bartosz Foroncewicz, Krzysztof Mucha, Radosław Zagożdżon, Zygmunt Pojda

**Affiliations:** 1 Laboratory of Cellular and Genetic Therapies, Center for Preclinical Research, Medical University of Warsaw, 02-097 Warsaw, Poland; katarzyna.zielniok@wum.edu.pl (K.Z.); mieszko.lachota@wum.edu.pl (M.L.); natalia.krata@wum.edu.pl (N.W.-K.); 2 Department of Regenerative Medicine, Maria Skłodowska-Curie National Research Institute of Oncology, 02-781 Warsaw, Poland; rusinek.kinga@outlook.com (K.R.); anna.slysz@nio.gov.pl (A.S.); ewa.baczynska@nio.gov.pl (E.B.); anna.szpakowska@nio.gov.pl (A.S.); martyna.ciepielak@nio.gov.pl (M.C.); zygmunt.pojda@nio.gov.pl (Z.P.); 3 Promix (ProteogenOmix in Medicine), Department of Clinical Immunology, Medical University of Warsaw, 02-006 Warsaw, Poland; bartosz.foroncewicz@wum.edu.pl (B.F.); krzysztof.mucha@wum.edu.pl (K.M.); 4 Department of Transplantology, Immunology, Nephrology and Internal Diseases, Medical University of Warsaw, 02-006 Warsaw, Poland; 5 Institute of Biochemistry and Biophysics, Polish Academy of Sciences, 02-106 Warsaw, Poland

**Keywords:** glioblastoma multiforme, GBM, chemokines, 3D-bioprinting, hypoxia, mesenchymal stromal cells, MSCs

## Abstract

3D bioprinting has become a valuable tool for studying the biology of solid tumors, including glioblastoma multiforme (GBM). Our analysis of publicly available bulk RNA and single-cell sequencing data has allowed us to define the chemotactic profile of GBM tumors and identify the cell types that secrete particular chemokines in the GBM tumor microenvironment (TME). Our findings indicate that primary GBM tissues express multiple chemokines, whereas spherical monocultures of GBM cells significantly lose this diversity. Subsequently, the comparative analysis of GBM spherical monocultures vs. 3D-bioprinted multicultures of cells showed a restoration of chemokine profile diversity in 3D-bioprinted cultures. Furthermore, single-cell RNA-Seq analysis showed that cells of the perivascular niche (pericytes and endocytes) express multiple chemokines in the GBM TME. Next, we 3D-bioprinted cells from two glioblastoma cell lines, U-251 and DK-MG, alone and as co-cultures with mesenchymal stromal cells (representing cells of the perivascular niche) and assessed the chemokine secretome. The results clearly demonstrated that the interaction of tumors and mesenchymal cells leads to in a significant increase in the repertoire and levels of secreted chemokines under culture in 21% O_2_ and 1% O_2_. Our study indicates that cells of the perivascular niche may perform a substantial role in shaping the chemokine microenvironment in GBM tumors.

## 1. Introduction

The development of anticancer immunotherapy and personalized treatment has created the need to implement advanced research tools that reproduce features of the tumor microenvironment (TME) under laboratory conditions [1]. Among those, 3D bioprinting is currently one of the most promising platforms to study in vitro the simulated complexity and dynamics of the TME as well as to create composites of tumor models from established cell lines and/or patient-derived components. This approach may allow us to provide personalized therapy screening in a relatively short time, limiting the use of animal models, as well as to provide a platform for a drug development and therapy target discovery [2]. Indeed, 3D-bioprinted models have become valuable tools for studying the biology of solid tumors, including the most aggressive and invasive primary brain tumor, glioblastoma multiforme (GBM). It has been postulated that intratumoral heterogeneity and the complex nature of the intricate TME communication contribute to the resistance of GBM cells to therapy and invasion [2]. The generation of 3D-bioprinted glioma models aims to more accurately reproduce the complex characteristics of GBM tissue compared to traditional 2D in vitro cell cultures and patient explanted ex vivo cultures in 3D spheroids. The GBM 3D-bioprinted models studied so far contain various cellular and non-cellular components, such as extracellular matrix (ECM) proteins, immune cells, nerve cells, and blood vessels, allowing the biology of GBM to be studied with all paracrine signaling interactions between glioblastoma cells and the surrounding microenvironment [2,3,4]. 

There are various approaches to 3D bioprinting models of GBM, usually aimed at reproducing their cellular composition and structure. However, little is known about the chemotactic landscape of both the GBM primary tumors themselves and their 3D-bioprinted models, so we address this subject in the current report. We have been particularly interested in investigating the area of secreted chemokines, since the modified secretion of chemotactic factors by GBMs affects tumor growth, invasion, angiogenesis, and reduced immune cell recruitment and modifies the function of TME-resident myeloid cells, ultimately limiting their sensitivity to immunotherapies (reviewed in [5]). However, the existing literature data usually relate to a specific chemokine-receptor axis or are review papers in which the collected and summarized data often concern different experimental systems. In the present study, we reanalyzed publicly available transcriptional data from RNA sequencing of GBM tumors, and based on these results, we focused our investigation on pericyte precursors, being cells with a potentially vital role in shaping the chemotactic profile of GBM.

Pericytes are a highly heterogeneous-in-origin group of cells embedded in the basement membrane of brain blood microvessels, located centrally between endothelial cells, astrocytes, and neurons in the neurovascular unit (NVU) [6]. Although there are many different views on the origin of brain pericytes (from yolk sac-derived macrophage progenitor cells, bone marrow progenitor cells, or phagocytic macrophages), it is generally accepted that, apart from forebrain pericytes, which are mainly derived from neural crest cells, pericytes in other parts of the brain are derived from mesodermal mesenchymal stromal cells (MSCs) [7]. However, due to the possession of a large number of overlapping markers and similar characteristics, there is an equally widespread view that pericytes are the progenitors of MSCs [8]. Regardless of the perspective taken, pericytes, likewise, mesenchymal stromal cells, show great plasticity in generating functional responses to microenvironmental stimuli that are critical for central nervous system function and are involved in maintaining the brain–immune interface [6,9]. In addition to their pluripotent progenitor cell properties [10,11,12,13], pericytes play a special role due to their paracrine properties to maintain the blood–brain barrier in collaboration with astrocytes, neurons, and endothelial cells and to promote the regeneration of the microenvironment by releasing nutritional factors and regulating the local immune response, serving a key role in maintaining brain homeostasis. However, their involvement in shaping the chemotactic secretome of the GBM remains to be elucidated. 

Chemokines modify the GBM microenvironment, participating in many tumor-related processes, affecting cell proliferation and growth, angiogenesis, vascular mimicry, and metastasis (reviewed in [14,15]). Therefore, chemokines become a promising target for the development of new immunotherapeutic and anti-angiogenic strategies for the treatment of GBM. They are involved not only in regulating the targeted migration of immune cells within the tumor [16,17], but due to the presence of chemokine receptors on the surface of GBM cells, they are also responsible for the migratory direction of glioma cells [15,18]. Moreover, studies have shown that the most common routes of GBM cell invasion involve perivascular space and white-matter tracts (reviewed in [19]). It should also be noted that in highly hypoxic tumors such as GBM (in which hypoxia reaches pO_2_ as low as 0.1% [20]), it is the availability of oxygen that determines not only the distribution and invasion pathways of tumor cells but also shapes their secretory activity, including the secretion of chemokines [21].

Therefore, we performed an in-depth analysis of transcriptional data from both TCGA and CGCA cohorts, primary GBM tissues with corresponding ex vivo tissue-derived cultures, 3D bioprinting, and single-cell RNA-Seq to describe a chemokine profile in glioblastoma multiforme and to indicate the cell types that might be involved in its formation. On this basis, we created a simplified 3D-bioprinted model of glioblastoma multiforme cells and pericyte precursors, MSCs, and analyzed the collected secretome by the Luminex method. We indicate how their mutual communication and bioprinting parameters shape the amount and type of secreted chemokines.

## 2. Materials and Methods

### 2.1. Bioinformatic Data Analysis

To determine the expression profile of chemokine genes in glioblastoma multiforme tumor tissues, publicly available sequencing data found in the TCGA and CGGA repositories were used. The gene expression profiles of FPKM (fragments per kilobase of transcript per million fragments mapped) type of glioblastoma multiforme from 166 patients with their clinical and survival data were downloaded from TCGA Xena Hub, cohort TCGA-GBM filtered for glioblastoma https://tcga.xenahubs.net (accessed on 9 February 2022) [22,23]. The gene expression profiles of FPKM type of glioblastoma multiforme from 663 patients and their clinical and survival data were downloaded from Chinese Glioma Genome Atlas http://www.cgga.org.cn/ (accessed on 9 February 2022) [24].

To compare chemokine gene expression in GBM tumor tissue and tumor-derived spherical cell cultures, we used sequencing data previously published by Gozdz et al. (2022) [25] and deposited in the European Genome–phenome Archive (EGA) under accession number EGAS00001006267.

RNA sequencing reads were processed using nf-core/rnaseq; they were aligned to the human genome (GRCh38) using the HISAT2 (v2.2.0) algorithm, then transcripts were assembled and quantified using StringTie2 (v2.1.7) [26,27,28]. For differential gene expression analysis, the RNA-Seq expression data were imported from StringTie using tximport (v1.28.0) [29]. Differential gene expression analysis was performed using DESeq2 (v1.40.2) [30]. To optimize power, we used Independent Hypothesis Weighting (IHW, v1.28.0) with an adjusted *p*-value threshold < 0.05 for reporting DEGs [31]. The source code for all the analyses will be provided upon request [32].

Sequencing data comparing chemokine expression in 2D, spherical, and 3D-bioprinted glioblastoma multiforme culture systems were utilized from the article by Tang et al. (2020) [3] and downloaded from the Gene Expression Omnibus (GEO) data repository at the accession number GSE147147. 

Single-cell sequencing data were used for UMAP analysis indicating chemokine gene expression in different cell types of the GBM tumor microenvironment. The single-cell gene expression profiles of 2 low-grade glioma and 16 glioblastoma multiforme patients (201,986 cells) were downloaded from Single Cell Portal (SPC1985/GSE182109) https://singlecell.broadinstitute.org/single_cell (accessed on 13 March 2024) [33]. Data were accessed as described above and imported into Seurat (v4.9.9.905) along with meta- and clustering data from the original article by Abdelfattah et al. [34,35]. Visualization was performed using Nebulosa (v1.10.0) [36]. The source code for all analyses will be available from the corresponding author upon request.

### 2.2. Glioblastoma Multiforme Cell Lines Culture

The human glioblastoma multiforme cell line U-251MG (RRID:CVCL_0021) was obtained from Merck Millipore (Darmstadt, Germany). U-251 cells were cultured in MEM Complete Medium, with NEAA, 1 mM sodium pyruvate, 2 mM L-Glut, and 10% FBS (all Merck Millipore), supplemented with 1% Penicillin-Streptomycin (Gibco™, Thermo Fisher Scientific, Waltham, MA, USA). The DK-MG cell line (RRID:CVCL_1173) was purchased from DSMZ-German Collection of Microorganisms and Cell Cultures GmbH (Braunschweig, Germany) and cultured in RPMI Medium 1640, supplemented with 1% Pen-Strep and 10% fetal bovine serum ( all Thermo Fisher Scientific, Waltham, MA, USA). Cells were cultured at 37 °C in a humidified atmosphere and 5% CO_2,_ until an optical confluence of 80%, detached with 0.25% trypsin-EDTA (Gibco™, Thermo Fisher Scientific, Waltham, MA, USA). The cultures were routinely tested for mycoplasma contamination.

### 2.3. Isolation of Mesenchymal Stromal Cells (MSCs) from Human Adipose Tissue

Pericyte precursor cells, MSCs, were chosen for our 3D-bioprinting study because they are considered a highly promising source for neural tissue engineering (i.e., they can be obtained in large quantities and tolerate the shear stresses of bioprinting relatively well) [37]. The choice of tissue source of MSCs was based on a study by Urrutia et al. on the differentiation of MSCs into neurons, which showed that MSCs derived from adipose tissue had a higher proliferation rate and increased expression of neuronal markers compared to MSCs derived from other tissues [38]. Although our study was not aimed at a regenerative approach, these results indicate that adipose tissue might be a good source of MSCs for GBM TME reconstruction.

The human adipose tissue was collected during a liposuction procedure from the superficial abdominal regions of healthy donors. Isolation of mesenchymal stromal cells from human adipose tissue was approved by the Bioethics Committee at the Maria Sklodowska-Curie National Research Institute of Oncology in Warsaw (Approval number: 33/2021). Informed consent was obtained from participants involved in the study. To remove red blood cells, the adipose tissue was mixed in a 2:1 (*v*/*v*) ratio with phosphate-buffered saline (PBS). Following phase separation, PBS containing red blood cells was discarded. The purification process was repeated three times. Then, to digest the tissue, a 0.075% (*w*/*v*) collagenase solution from *Clostridium histolyticum* (Merck Millipore, Darmstadt, Germany) in PBS was added to the adipose tissue (1:2), incubated at 37 °C for 1.5 h, and shaken at 15-min intervals. The digestion process was stopped by adding human albumin at a final concentration of 2% (*v*/*v*). The mixture was centrifuged at 400× *g* for 10 min at room temperature. Liquid fat and salt interphases were discarded, and the pellet was resuspended in PBS. The cell suspension was filtered through a 100 µm nylon mesh strainer, washed in PBS, and centrifuged (400× *g*) for 10 min at room temperature. Cell number and viability were determined, and then they were seeded into plastic flasks in DMEM (Dulbecco′s Modified Eagle′s Medium; Merck Millipore, Darmstadt, Germany) supplemented with 10% FBS (Thermo Fisher Scientific, Waltham, MA, USA) and 1% Penicillin-Streptomycin (Pen-Strep; Gibco™, Thermo Fisher Scientific, Waltham, MA, USA). Cells were cultured at 37 °C in a humidified atmosphere and 5% CO_2_ until an optical confluence of 80% then were detached with 0.25% trypsin-EDTA (Gibco™, Thermo Fisher Scientific, Waltham, MA, USA). After the cells were identified as previously described [39], they were then used at a third passage for further experiments. In order to determine the response of glioblastoma multiforme cell lines to the experimental parameters used without introducing factors related to the individual variability of MSCs from different donors, we chose to use MSCs isolated from the same donor in all the 3D-bioprinting experiments.

### 2.4. 3D Bioprinting

**Figure 1:**
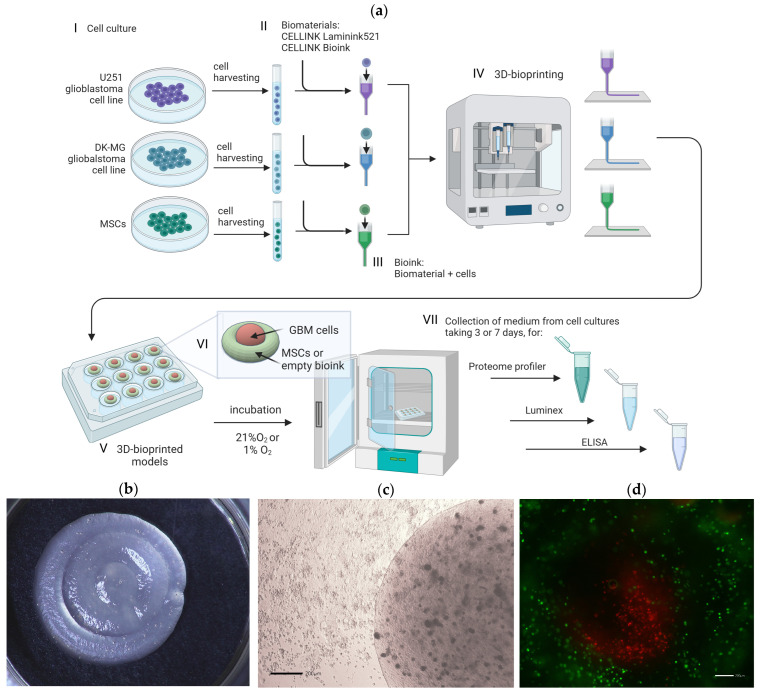
(**a**) Schematic diagram of in vitro 3D bioprinting of glioblastoma multiforme cell lines with mesenchymal stromal cells with the experiment workflow: (I) Cell culture of glioblastoma multiforme cell lines U-251 and DK-MG and human mesenchymal stromal cells (MSCs); (II) Preparation of CELLINK Laminink521 and CELLINK Bioink hydrogels; (III) Preparation of bioink by mixing hydrogels with cells; (IV) Bioprinting process with BIO X™ bioprinter (CELLINK, Bico Group, Gothenburg, Sweden); (V) Culture of 3D-bioprinted models; (VI) Scheme of 3D-bioprinted model design. Red area: U-251 cells, DK-MG cells, or bioink without cells; green area: MSCs or bioink without cells, accordingly. (VII) Medium collection for further experiments from at least 3 culture replicates. A detailed description is provided in the text, Section 2.4 3D Bioprinting. Created with biorender.com; (**b**) Representative photography of 3D bioprint; (**c**) Representative light microscopy image of 3D bioprint; scale bar 200 µm (**d**) Representative fluorescence microscopy image of a 3D bioprint with the sheath core of glioma cell line U-251 surrounded by MSCs. Green signal: MSCs labeled with PKH67, red signal: U251 labeled with PKH26. Scale bar 200 µm.

We used CELLINK Laminink521 and CELLINK Bioink biomaterials provided by Cellink (Bico Group; Gothenburg, Sweden) to create 3D-bioprinting models (a schematic overview of the 3D-bioprinting experiments is shown in Figure 1a). First, U-251, DK-MG, or human mesenchymal stromal cells (MSCs) were resuspended separately in PBS (Figure 1a(I)), added to CELLINK Laminink521 and CELLINK Bioink (Figure 1a(II)), and mixed according to the manufacturer’s instructions (‘Mixing Cells Protocol’ CELLINK Series) (Figure 1a(III)). Cells were prepared at a final density of 1.2 × 10^6^/mL of bioink. The bioinks containing cells were transferred into an empty 3 mL sterile cartridge (Cellink, Bico Group, Gothenburg, Sweden), and used for 3D bioprinting (Figure 1a(IV)). 

### 2.5. Bioprinting of 3D Glioblastoma Multiforme Constructs

The 3D constructs of bioinks with glioblastoma multiforme cell lines, U-251 and DK-MG, and human mesenchymal stromal cells were biofabricated using an extrusion-based 3D bioprinter BIO X™ (Cellink, Bico Group, Gothenburg, Sweden). Prepared 3 mL cartridges were equipped with 22 G sterile conical bioprinting nozzles (Cellink, Bico Group, Gothenburg, Sweden), placed within the pneumatic printhead, and connected to the tubing. The STL files with the architecture of a 3D model were generated using Autodesk Tinkercad (Figure 1a(VI)). All constructs were printed into a 24-well plate (Falcon, Becton Dickinson, Franklin Lakes, NJ, USA), with a pressure ranging from 13–23 kPa depending on the usage of bioink and cell line types. The bioprinted constructs were crosslinked for 1.5 min with CaCl_2_ Crosslinking Agent (50 mM) provided by Cellink (Gothenburg, Sweden). The 3D-bioprinted constructs were cultured for 72 or 168 h in 800 µL of DMEM supplemented with 4% FBS at 37 °C in 1% O_2_ (hypoxia) or 21% O_2_.

### 2.6. Collection of Samples and Chemokine Profiling

The 3D bioprints were incubated in 21% O_2_ or 1% O_2_ for 3 and 7 days, after which the culture medium from each well was collected into 1.5 mL microcentrifuge tubes (Thermo Scientific™, Waltham, MA, USA) and centrifuged for 5 min at 4500 rpm. The 700 µL of each supernatant was collected, and the material was stored at −80 °C until the Proteome Profiler Chemokine Array, Luminex Multiplex Assay and Enzyme-Linked immunosorbent assay (ELISA) were performed. On the day of analysis, the collected medium was thawed on ice and directly used for analysis. Samples were only thawed once and not refrozen. To initially profile the chemokines secreted by GBM 3D-bioprinted cultures, we performed dot blot screening using the Proteome Profiler Human Chemokine Array Kit (cat. #ARY017, R&D Systems, Bio-Techne, Minneapolis, MN, USA). For this purpose, samples cultured on CELLINK Laminink from 4 experimental replicates were pooled into 1 sample just prior to analysis. We analyzed 5 samples (U251, DK-MG, MSCs, U251 + MSCs, and DK-MG + MSCs) each from a 3-day culture in 21% O_2_ and 5 samples cultured in 1% O_2_. In addition, chemokine levels in growth medium incubated in an empty bioink under the same conditions were also examined. We performed the assay according to the manufacturer’s protocol. The chemiluminescence of the membranes was read using Chemidoc MP (Bio-Rad Laboratories, Hercules, CA, USA), and the integrated optical density of each dot was measured and quantified using Image Lab software (Bio-Rad Laboratories, Hercules, CA, USA).

### 2.7. Luminex

Quantitative assessment of chemokine levels was performed on a Luminex200 Instrument System (Luminex Corporation, Austin, TX, USA) using xPONENT Basic Plus software (v. 3.1, Luminex Corporation, Austin, TX, USA) and the Human Luminex Discovery Assay kit (#LXSAHM, R&D Systems, Bio-Techne, Minneapolis, MN, USA). Luminex custom kits comprising CXCL16, CCL5, CCL7, CCL2, CXCL8, CCL3, CCL4, chemerin, CX3CL1, CXCL1, CXCL5, and CXCL9 were purchased for analysis. Analytes were selected based on the results of initial profiling using the Proteome profiler. Briefly, all reagents were first brought to room temperature and prepared according to the manufacturer’s instructions. Then, 50 µL of standard or undiluted sample was added per well to a 96-well plate. Next, 50 µL of microparticle cocktail was added to all wells. The plate was incubated for 2 h on a microplate shaker set at 800 rpm at room temperature. Then, the plate was placed on a magnet and washed three times. After washing, a biotin-antibody cocktail was added to each well and incubated for 1 h at room temperature on a shaker set at 800 rpm. Once the incubation was complete and the plate was washed three more times, 50 µL of Streptavidin-PE was added to each well. The plate was then incubated for 30 min at 800 rpm. The plate was then washed three times, and the microparticles were finally resuspended by adding 100 µL of wash buffer to each well. After 2 min of incubation, the plate was read on a Luminex200 system set according to the parameters described by the manufacturer. Analyses were performed on at least three experimental replicates, each in duplicate. The results were normalized by chemokine level values obtained in growth medium derived from parallel 3- or 7-day incubations of culture medium in a 3D-bioprinted blank bioink. Raw data were analyzed using Belysa^®^ Immunoassay Curve Fitting Software Version 1.2 (Merck Millipore, Darmstadt, Germany). 

### 2.8. ELISA

As CXCL12 was not available in the Human Luminex Discovery Assay kit, we decided to analyze its levels with a commercial ELISA kit. The Human CXCL12/SDF-1 alpha Quantikine ELISA Kit (#DSA00, R&D Systems, Bio-Techne, Minneapolis, MN, USA) was used to quantify CXCL12 levels. Tests were performed according to the manufacturer’s protocol. Analyses were performed on at least three experimental replicates, each in duplicate. The results were normalized by CXCL12 level obtained in growth medium derived from parallel 3- or 7-day incubations of culture medium in a 3D-bioprinted blank bioink.

### 2.9. Statistical Analysis

The chemokine levels obtained from Luminex and ELISA analyses are expressed as means on heatmap graphs. Statistical significance was determined using a mixed-effects model analysis with Geisser–Greenhouse’s correction in GraphPad Prism software (version 10.3.0, GraphPad Software, Boston, MA, USA). This mixed model used a compound symmetry covariance matrix, fitted using Restricted Maximum Likelihood (REML), assessing the variance of samples according to cell type (U251, DK-MG, MSCs, U251 + MSCs, and DK-MG + MSCs) and culture parameters (type of bioink, cell culture time, and oxygen content of the cell culture atmosphere). Cell means were subjected to a post hoc Tukey–Kramer multiple comparison test; *p* < 0.05 was considered statistically significant. * indicates *p*-value < 0.05, ** *p*-value < 0.01, *** *p*-value < 0.001, **** *p*-value < 0.The results of the analysis can be found in Supplementary Table S1.

## 3. Results

### 3.1. Gene Expression Analysis from the TCGA and CCGA Cohorts Revealed a Broad Chemokine Expression Profile in GBM Tissues

**Figure 2:**
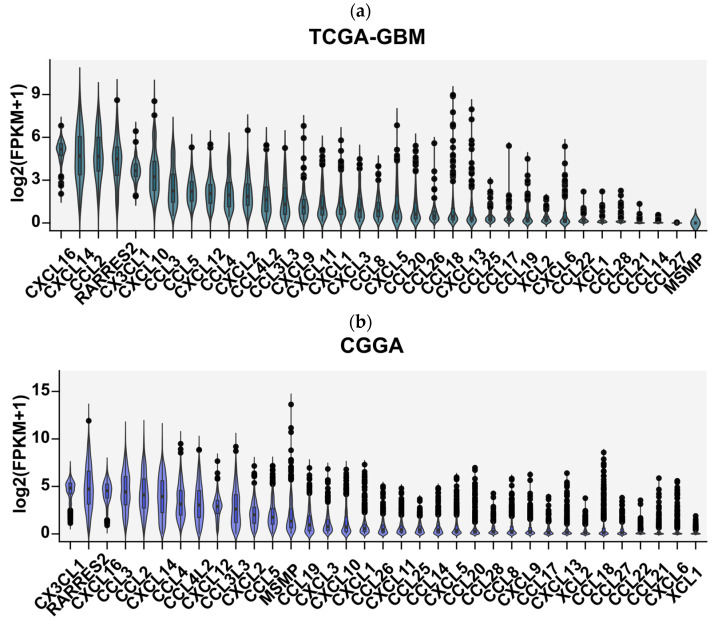
Expression profile of 35 chemokines ranked from most highly to least-expressed genes in glioblastoma multiforme tumor tissues obtained from analysis of RNA sequencing data from (**a**) TCGA-GTEx cohorts and (**b**) CGGA cohort. The gene expression profiles of FPKM (fragments per kilobase of transcript per million fragments mapped) type of glioblastoma multiforme samples of 166 patients were downloaded from TCGA Xena Hub cohort filtered for glioblastoma, accession date 9 February 2022 [22,23] and from 663 patients from Chinese Glioma Genome Atlas (http://www.cgga.org.cn/), accession date 9 February 2022 [24]. The graph shows normalized chemokine gene expression as log2-transformed FPKM + 1 values.

We first focused on determining the diversity of chemokines expressed in glioblastoma tissues. Analysis of over 800 tumor samples from the TCGA and CGGA cohorts revealed a consistent, broad chemokine expression profile in GBM, with *CXCL16*, *CX3CL1*, *CXCL14*, *RARRES2*, *CCL2*, *CXCL8*, *CXCL10*, CCR5 ligands (*CCL3*, *CCL4*, *CCL5*, *CCL3L3*, and *CCL4L2*), and *CXCL12* as the most highly expressed (Figure 2).

### 3.2. Glioblastoma Multiforme Primary Cell Cultures Show a Markedly Reduced Chemokine Expression Profile Compared to the Tumor Tissues from Which They Originated

**Figure 3:**
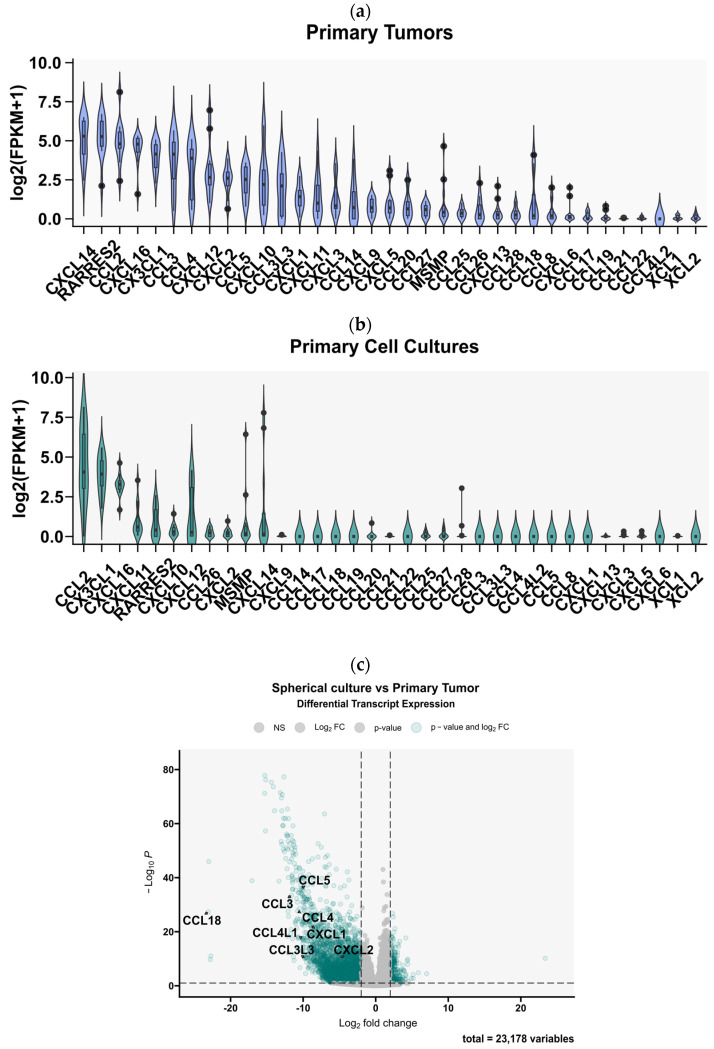

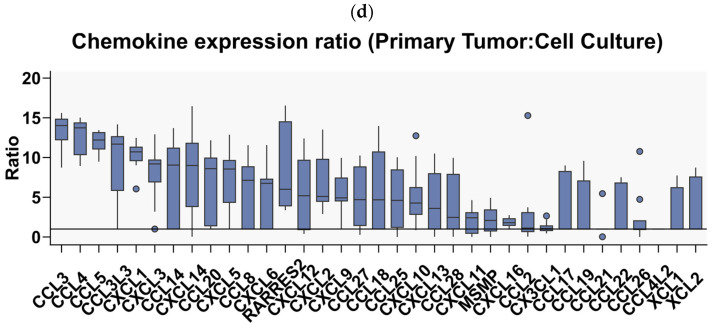
Comparative analysis of chemokine gene expression between glioblastoma multiforme tumor tissues and spherical cell cultures derived from these tumors. Chemokine gene expression profile obtained by analysis of RNA-Seq data from the article by Góźdź et al. (2022) [13] in (**a**) 12 primary tumors and (**b**) 12 primary spherical cell cultures derived from these tumors and maintained in 21% O_2._ The graph shows normalized chemokine genes expression as log2-transformed FPKM + 1 values. (**c**) A volcano plot of differential gene expression analysis between tumor tissues and cell cultures derived from these tissues. Points marked in green indicate genes with a statistically significant change in expression. The analysis showed significantly downregulated expression of 8 chemokine genes (marked in the graph) in spherical cultures compared to primary tumors. (**d**) A graph showing the ratio of chemokine gene expression in primary cultures versus expression in the tissues of the tumors from which they were derived, ranked from chemokine genes with the most to those with the least difference in expression.

Having determined the profile of expressed chemokines in GBM tissues, we then proceeded to investigate whether this profile is preserved in ex vivo cell cultures derived from the tumors. We used previously collected sequencing data comparing the chemokine expression profiles between 12 GBM tumor tissues and 12 ex vivo spheroid cultures derived from these tumors, which were maintained in serum-free neurobasal medium [25]. The results clearly demonstrated that the expression of many chemokines was significantly downregulated in GBM spheroid cultures (Figure 3a). In particular, the expression of *CCL3*, *CCL4* (and their ligands *CCL4L1* and *CCL3L3*), *CCL5*, *CXCL1*, *CXCL3*, *CCL14*, *CCL20*, *CXCL5*, *CCL8*, *CXCL6*, *CXCL12*, and *CXCL9* was significantly decreased (Figure 3a). None of the chemokines were upregulated in the GBM cells maintained in spheroid cultures. Moreover, the expression of only a few of the entire profile of chemokines expressed in GBM tumors was preserved in spherical cultures (mainly *CCL2*, *CX3CL1*, *CXCL16*, *CXCL11*, *CXCL10*, *CXCL12*, *CXCL26*, *CXCL2*, and *CXCL14*, Figure 3b). Within the most highly expressed chemokines in tumor tissues, we observed the largest decrease in the number of transcripts (indicated by the tumor/cell culture ratio) for *CCL3* (and *CCL3L3*), *CCL4* (and *CCL4L1*), *CCL5*, *CXCL1*, *CXCL3*, *CXCL14*, and *CXCL12,* whereas the smallest changes were observed for *CX3CL1*, *CCL2*, *CXCL16*, and *CXCL11* (Figure 3c).

### 3.3. 3D Bioprinting of Tumor-Derived Primary GBM Cells with Cells of the Tumor Microenvironment Partially Restores the Diversity of Expressed Chemokines

**Figure 4:**
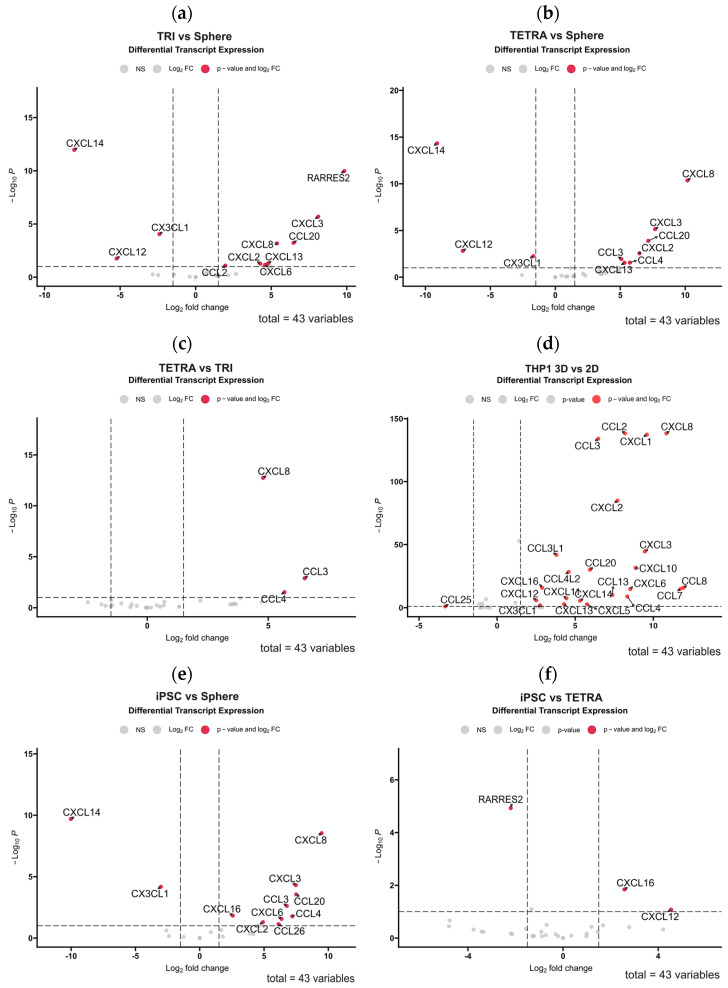
Volcano plot diagrams showing the results of differential chemokine gene expression analysis among different in vitro culture systems performed using RNA sequencing data previously published in Tang et al. (2020) [3]. The diagrams illustrate how the use of different 2D culture systems and 3D-bioprinted cultures of primary glioblastoma multiforme stem cells (GSCs) without/with astrocytes, neural progenitor cells (hNP1) and macrophages from THP1 lineage or differentiated from human-induced pluripotent stem cells (iPSCs), affects chemokine gene expression. Sphere culture: cell culture of GSCs in the form of non-adherent spheroids; TRI (triculture system): 3D-bioprinted culture of GSCs with surrounding astrocytes and hNP1; TETRA (tetraculture system): 3D-bioprinted culture of GSCs and macrophages from THP-1 lineage along with surrounding astrocytes and hNP1; iPSC system: 3D-bioprinted culture of GSCs along with macrophages differentiated from hiPSCs with surrounding astrocytes and hNPRed points in the graphs represent chemokine genes with a statistically significant difference in expression between the tested groups (downregulated if their Log_2_ fold change is below 0 or upregulated if it is above 0). The axes indicate the statistical significance (−Log_10_ *p*-value) versus fold change in expression (Log_2_ fold change) of significantly enriched chemokine genes in the comparative analysis of (**a**) TRI system to spherical culture, (**b**) TETRA system to spherical culture, (**c**) TETRA system to TRI system, (**d**) THP1 cell line culture in 3D bioprinting versus 2D culture, (**e**) iPSC system to spherical culture, and (**f**) iPSC system to TETRA system. Gene expression of 43 chemokines was analyzed in each comparison.

In the next step, we assessed whether the use of 3D-bioprinting technology of GBM cell co-culture with cells from the tumor microenvironment restores the chemokine expression profile of the GBM tumor. Using publicly available RNA sequencing data from the study by Tang et al. (2020) [3], we performed differential gene expression profiling for chemokines in various 3D-bioprinting systems using glioblastoma multiforme stem cells (GSCs, which are analogous to the ex vivo cultures described above). This system was based on glioblastoma multiforme cells collected from patients and cultured in serum-free neurobasal medium in combination with cells mimicking those found in the GBM microenvironment, which were macrophage-differentiated M2 monocytes of the THP-1 line, human neural progenitor cells (hNP1), astrocytes, and macrophages differentiated from hiPSCs (human-induced pluripotent stem cells). In this model, astrocytes and neural stem cells were 3D-bioprinted in an outer ring surrounding a core consisting of primary GBM cells cultured solo (triculture system, TRI) or with the addition of THP1 macrophages (tetraculture, TETRA). The bioinformatic analysis we performed clearly indicated differences in the profile of chemokines expressed for patient-derived GBM cells cultured solo in spheres compared to a both 3D-bioprinted systems in which the same GBM cells were cultured with other cell types of the tumor microenvironment (Figure 4a,b). The 3D-bioprinted culture of GBM cells resulted in significantly increased expressions of *RARRES2* (chemerin), *CXCL8*, *CXCL3*, *CCL20*, *CXCL2*, *CCL3*, *CCL4*, and *CXCL13* with a concomitant decrease in the expressions of *CXCL14*, *CXCL12*, and *CX3CL1* compared to those in the spheroid culture. Notably, the increased expression of only *CXCL8*, *CCL3*, and *CCL4* was associated with the presence of THP1 macrophages in the 3D-bioprinted culture, as shown in the comparison of the TRI versus TETRA culture (Figure 4c). This effect was relatively minor, considering how immense the effect of 3D bioprinting was on the diversity of chemokine expression for THP1 macrophages alone compared to their 2D culture (Figure 4d). A similar differential gene expression profile as in the comparison of TETRA system to spheroids emerged from the comparison of chemokine expression in the 3D-bioprinted TETRA system with iPSC-derived macrophages compared to GBM spheroids (Figure 4e). The differences were that *CXCL12* was not downregulated, while *CXCL16* and *CXCL6* were upregulated (Figure 4f shows the comparison of the iPSC-derived system to the TETRA system). The use of iPSC-derived macrophages resulted in the upregulation of *CXCL12* and *CXCL16* genes, but the *RARRES* was downregulated in this system.

### 3.4. Single Cell RNA-Seq Data Enabled Identification of the Cellular Source of Expressed Chemokines in GBM Tumors

**Figure 5:**
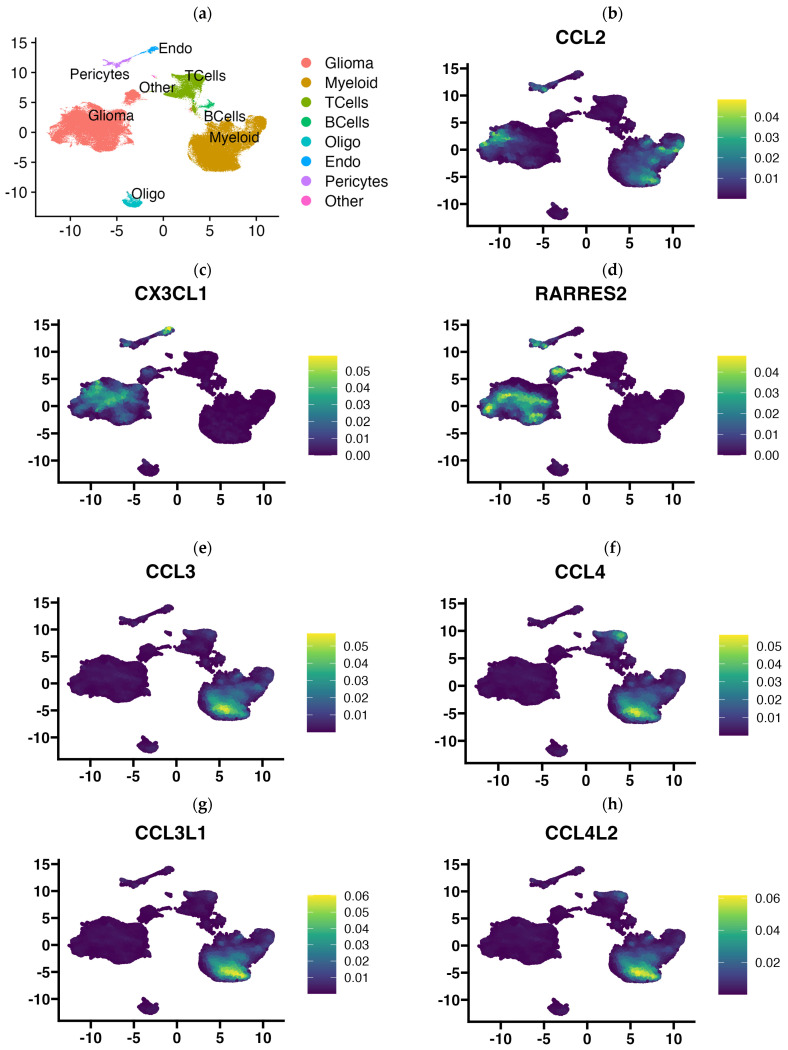

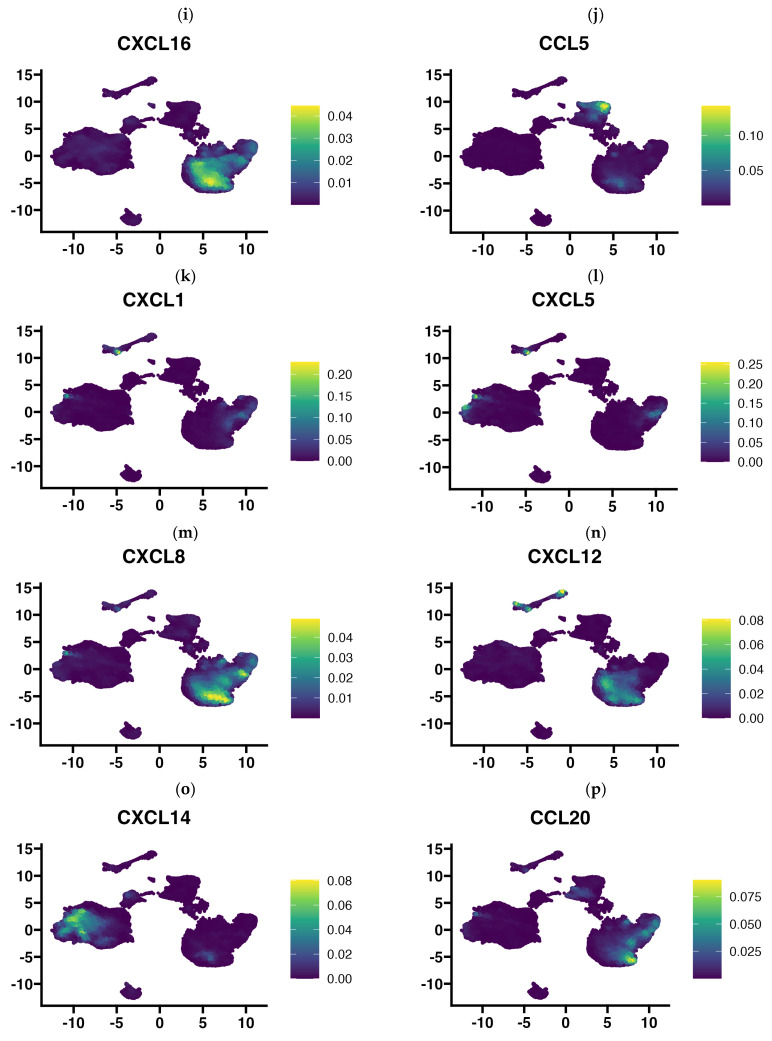

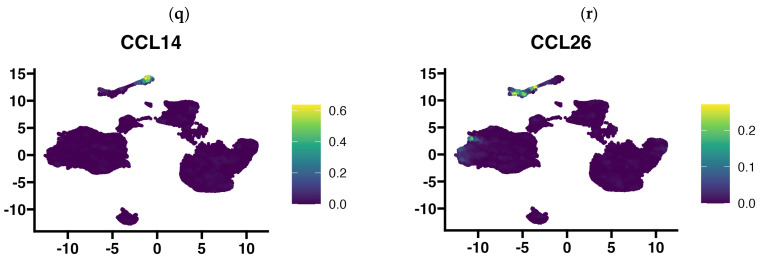
UMAP plots showing chemokine gene expression level according to the cell type present in glioblastoma multiforme tissues that were subjected to single-cell RNA-Seq analysis. Chemokine expression at a single-cell level from 2 low grade glioma and 16 primary glioblastoma multiforme tumors (201,986 cells) was projected onto the tSNE (t-distributed stochastic neighbor embedding) plots, annotated according to the cell signature clustering data from the original article by Abdelfattah et al. [34,35]. Diagram (**a**) indicates where certain cell types are located on the diagram: glioma cells, myeloid cells, T lymphocytes, B lymphocytes, oligodendrocytes, endocytes and pericytes, and others. Diagrams of the expression of (**b**) CCL2, (**c**) CX3CL1, (**d**) RARRES2, (**e**) CCL3, (**f**) CCL4, (**g**) CCL3L1, (**h**) CCL4L2, (**i**) CXCL16, (**j**) CCL5, (**k**) CXCL1, (**l**) CXCL5, (**m**) CXCL8, (**n**) CXCL12, (**o**) CXCL14, (**p**) CCL20, (**q**) CCL14, (**r**) CCL26, clustered according to cell type are presented above. Expression diagrams for the remaining chemokines are provided in Supplementary Figure S1. Single-cell RNA-Seq data were downloaded from Single Cell Portal (https://singlecell.broadinstitute.org/single_cell), accession date 13 March 2024 (SPC1985/GSE182109) [33] and imported into Seurat (v4.9.9.905). Visualization was performed using Nebulosa (v1.10.0) [36].

In view of the finding that the use of 3D-bioprinting technology using three types of cells of the tumor microenvironment did not reflect the full picture of chemokine expression that is found in GBM tumors, in the next step, we aimed to determine exactly which cells in the tumor tissue express particular chemokines. Using publicly available single-cell RNA-Seq data, we performed an analysis of chemokine expression in cells of the GBM microenvironment classified based on gene signature into several major cell types: glioma cells, myeloid cells (comprising monocytes, macrophages, dendritic cells, and granulocytes), T lymphocytes, B lymphocytes, oligodendrocytes, endocytic cells, pericytes, and others (Figure 5a). Our results indicate that the rich profile of expressed chemokines in GBM tumors is contributed to by all cell types of the tumor microenvironment, including pericytes and endothelial cells. Among the most prominently expressed chemokine genes in GBM tissue, *CCL2* is the chemokine gene that was expressed by myeloid cells, tumor cells, and pericytes (Figure 5b). *CX3CL1* was expressed in tumor cells, pericytes, and prominently in endothelial cells, while having negligible expression in immune cells (myeloid cells and T and B lymphocytes, Figure 5c). Similarly, the *RARRES2* gene (encoding chemerin) was highly expressed among various tumor cells and pericytes but was not clearly expressed by endothelial cells (Figure 5d). Conversely, *CCL3*, *CCL4*, *CCL3L3*, *CCL4L1*, and *CXCL16* genes were mainly expressed by immune cells (myeloid and T cells), but trace amounts of their expression were also detected in other cell types (Figure 5e–i). *CCL5* appears to be exclusively restricted to expression in T lymphocytes and myeloid cells (Figure 5j). The genes of the next chemokines, *CXCL1,-2,-3,-5,-6,-8*, have a very similar expression pattern (Figure 5k–m and Supplementary Figure S1), which includes a very restricted subpopulation of glioma cells, pericytes, and myeloid cells (among the latter, *CXCL2,-3,-8* are highly expressed). *CXCL12* was highly expressed in pericytes, endothelial and myeloid cells, and, at a very minimal level, also in glioma cells. *CXCL14* was expressed mainly by a diverse group of glioma cells, while *CCL20*, in contrast, was mainly expressed by myeloid cells and T lymphocytes. A very cell-type-restricted expression was found for *CCL14* (which was expressed almost exclusively in endothelial cells and a little in pericytes, Figure 5q) and *CCL26* (expressed in pericytes and a small group of glial cells, Figure 5r). Of the 44 chemokines investigated in our analysis, pericytes expressed, to a greater or lesser extent, the genes of more than half of them (Figure 5 and Supplementary Figure S1).

### 3.5. The Use of 3D-Bioprinted Co-Cultures of GBM and Mesenchymal Cells Reveals the Profound Effect of Cell–Cell Interactions, Type of Biomaterial, and Oxygen Partial Pressure on Chemokine Secretion

**Figure 6:**
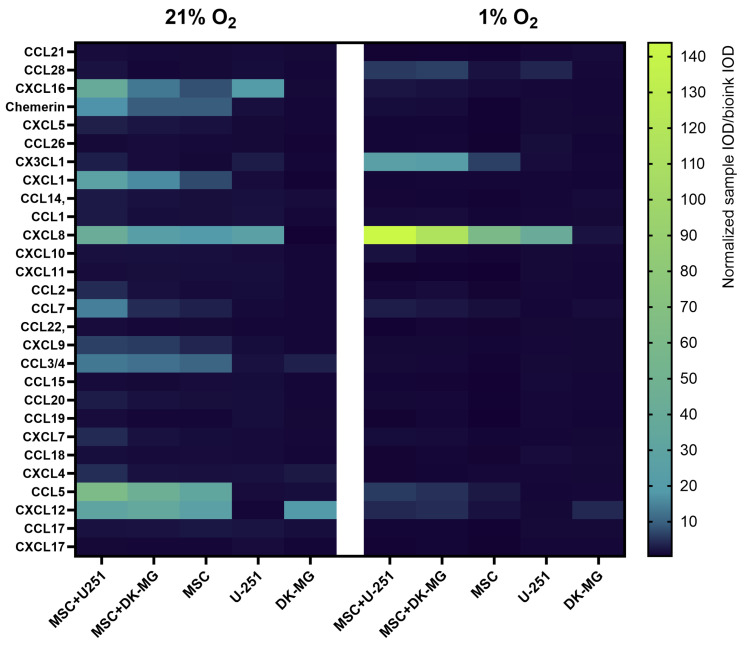
Heatmap showing relative chemokine secretion levels in medium collected from glioblastoma multiforme U251 and DK-MG cell lines 3D-bioprinted on CELLLINK Laminink521 with/without mesenchymal stromal cells (MSC) and cultured for 3 days in 21% O_2_ and 1% O_2_. Profiling was performed using the Proteome Profiler Human Chemokine Array Kit (R&D systems, Bio-Techne, Minneapolis, MN, USA). Samples pooled from 4 experimental replicates were used for profiling. The values on the graph represent the ratio of normalized optical density of dot blots from the sample to the optical density of dot blots from the culture medium incubated for 3 days with empty bioink.

In view of how many of the chemokines in GBM tissues appeared to be expressed by cells of the vascular niche (pericytes and endothelial cells), and given that microglia and myeloid cells are much more frequently used for studies in 3D-bioprinted systems than perivascular cells, in the next step, we created a simplified 3D-bioprinted model based on the co-culture of two GBM cell lines with cells of mesenchymal origin. We aimed to determine if and how the secretion of selected chemokines by glioblastoma cell lines U251 and DK-MG is affected by the presence of paracrine signals from mesenchymal stromal cells (MSCs), the type of biomaterial used, the volumetric oxygen content [vol%] of the cell culture atmosphere, and the time the cells are cultured. As a preliminary step, we performed chemokine profiling using Proteome Profiler in pooled samples from four experimental replicates (bioprinted on Laminink521) to select chemokines for quantitative analysis by Luminex. The results clearly indicated that cultures in 21% O_2_ showed a substantially richer chemokine secretion profile than those in 1% O_2_ (Figure 6). The glioblastoma cell lines U251 and DK-MG secreted very few chemokines in 21% O_2_ cultures, mainly CXCL16, CX3CL1, and CXCL8 for U251 cells and CCL3/4 and CXCL12 for DK-MG cells, and this profile was even lower in 1% OA more extensive profile of secreted chemokines characterized MSCs, but the highest signal indicative of chemokine secretion was found in co-cultures (especially U251 with MSCs). The only chemokines with signal levels that increased with decreasing oxygen were CXCL8 and CX3CLWhile the signal levels of CCL7, CCL5, and CXCL12 secreted by co-cultures decreased in 1% O_2_ compared to those in 21% O_2_, the signal of other chemokines (CXCL16, Chemerin, CXCL1, CCL2, CXCL9, CCL3/4, CXCL7, and CXCL4) almost completely faded in 1% OThe chemokine that was not secreted in 21% O_2_ but appeared in the secretome of cells cultured in 1% O_2_, according to the signal level in Proteome Profiler, was CCL28.

**Figure 7:**
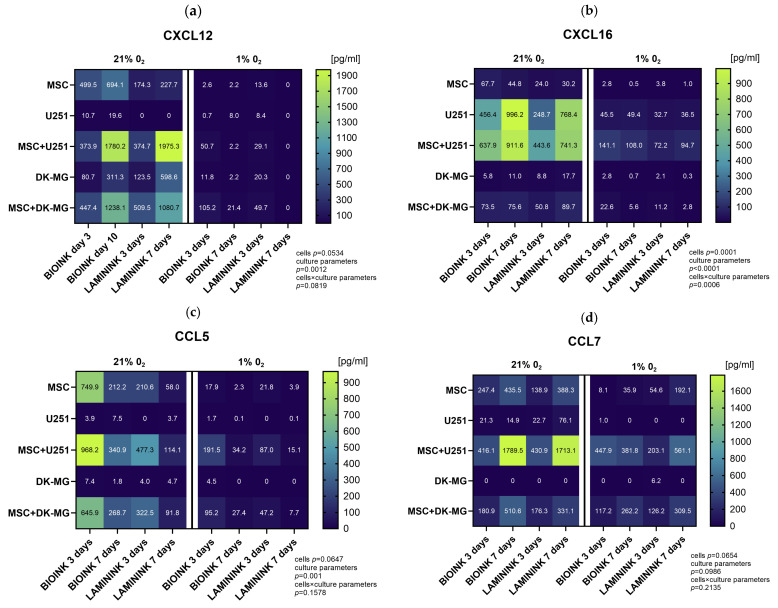

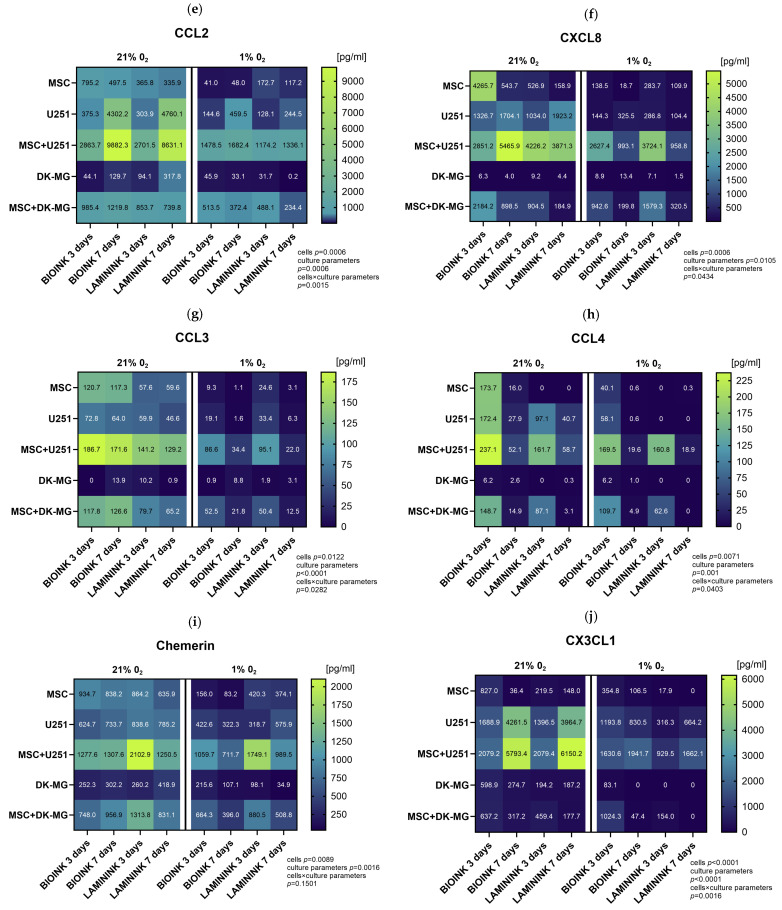

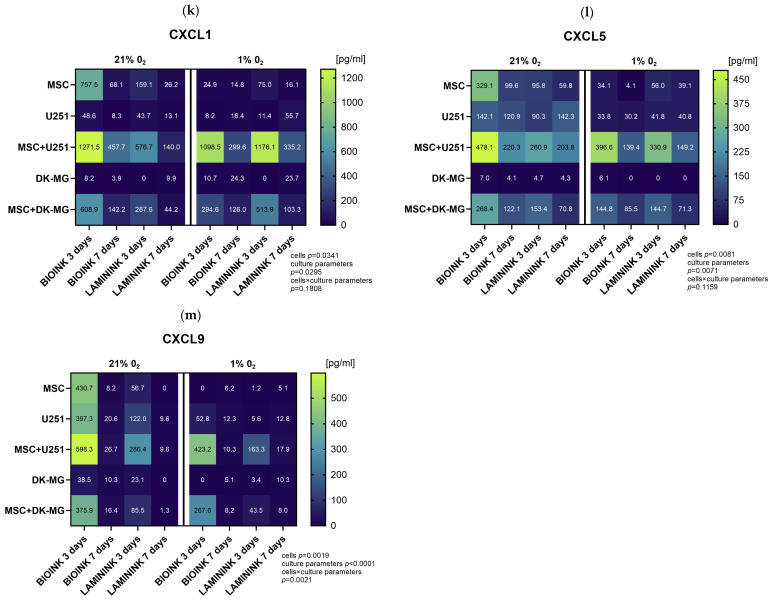
Heatmap plots showing the mean levels of selected chemokines in the medium from 3D-bioprinted cultures of glioblastoma multiforme cell lines. Quantitative evaluation of (**a**) CXCL12, (**b**) CXCL16, (**c**) CCL5, (**d**) CCL7, (**e**) CCL2, (**f**) CXCL8, (**g**) CCL3, (**h**) CCL4, (**i**) chemerin, (**j**) CX3CL1, (**k**) CXCL1, (**l**) CXCL5, and (**m**) CXCL9 secretion by Luminex method in 3D-bioprinted cultures of U251 and DK-MG glioblastoma multiforme cell lines with/without mesenchymal stromal cells (MSC), maintained in 21% O_2_ and 1% O_2_ on two types of bioink, CELLLINK Bioink or CELLLINK Laminink521, collected after 3 and 7 days of culture. Values were normalized by the chemokine levels in the culture medium maintained for 3 or 7 days with empty bioink. The levels of chemokines were determined in [pg/mL]. The graph shows numerical averages from at least three experimental replicates for each condition. Statistical significance was determined using a mixed-effects model analysis with Geisser–Greenhouse’s correction in GraphPad Prism software (version 10.3.0). The detailed results of the multiple comparisons of the mixed-effects model analysis can be found in Supplementary Table S1.

In order to precisely quantify chemokine secretion in the created 3D-bioprinting model of glioma–mesenchymal cell interactions, we further assessed the cell culture secretomes using the Luminex method. In this part, we analyzed cells bioprinted on two biomaterials, CELLINK Laminink521 and CELLINK Bioink. The results of the quantitative assessment in general confirmed the previous profiling, providing evidence of diminished chemokine secretion under culture conditions of 1% O_2_ compared to 21% O_2_ (Figure 7). The chemokines with the highest decrease in secretion levels in 1% O_2_ compared to 21% O_2_ were CXCL12 (Figure 7a), CXCL16 (Figure 7b), CCL5 (Figure 7c), and CCL7 (Figure 7d). However, as opposed to Proteome Profiler, quantitative analyses indicated that the vast majority of the chemokines secreted under 21% O_2_ are still secreted at 1% O_2_, and in the cases of CXCL1 (Figure 7k), CXCL5 (Figure 7l), chemerin (Figure 7i), and CX3CL1 (Figure 7j) for the co-culture, at entirely comparable levels. Furthermore, the results demonstrated that there are large differences in the secretion levels of different chemokines, as well as in the secretion levels of the same chemokine between the two glioblastoma cell lines. The chemokines secreted at the highest levels in our 3D-bioprinted system were CCL2 (up to 9000 pg/mL, Figure 7e), CXCL8 (up to 5000 pg/mL, Figure 7f), CX3CL1, (up to 6000 pg/mL, Figure 7j) and chemerin (up to 2000 pg/mL, Figure 7i), while CCL3 (Figure 7g) and CCL4 (Figure 7h) were secreted at the lowest levels, which did not exceed 200 pg/mL. For almost every chemokine tested, the highest levels were found in GBM co-cultures at both 21% O_2_ and 1% O_2_, and due to the generally higher levels of chemokine secretion by the U251 cell line than those by DK-MG, these were U251 cultures with MSCs. The results indicated that the source of CCL5, CCL7, and CXCL1 in our experimental system was mainly MSCs, while CXCL16 was predominantly secreted by glioblastoma U251 cells. The effect of the biomaterial itself on chemokine secretion in 3D-bioprinted cultures is also noteworthy. Although the analysis shows that it has a greater effect on the secretion of chemokines by MSCs (for which CELLINK Bioink is advantageous for almost every tested chemokine), differences can also be observed for GBM cell lines. CXCL16 was secreted by U251 in higher amounts when cultured on Bioink than on Laminink521 both solo and in co-culture, whereas DK-MG secreted more CXCL12 on Laminink521 than on Bioink. However, the differences in the secretion of most chemokines in the co-culture system seem to be related to the effect of the biomaterial on MSCs rather than that on glioblastoma cells. Finally, the secretion of chemokines varied greatly across culture times. When cultured under 21% O_2_, levels of CXCL12, CXCL16, CCL7, CCL2, and CX3CL1 in the culture medium accumulated significantly over time, while levels of CCL5, CCL4, CXCL1, CXCL5, and CXCL9 were significantly lower after 7 days of culture. Remarkably, at 1% O_2_ culture, the levels of secreted chemokines by GBM-MSC co-cultures were largely preserved, even when in solo GBM and MSC cultures, they decreased significantly compared to in 21% O_2_.

## 4. Discussion

Immune cell migration among solid tumors even of a single type is highly unpredictable due to their ectopicity, heterogeneity, and differences in anatomy [16]. Understanding the chemotactic microenvironment of tumor tissue, however, is crucial to uncover and remove immune cell entry blockages, especially with regard to current cellular adoptive therapies [16]. While chemokines play an essential role in directing the migration of effector cells of the anti-tumor response, they are also abundantly utilized by tumor cells to attract circulating cells (such as myeloid cells) to create a locally highly immunosuppressive and pro-malignant microenvironment that promotes tumor growth and serves as one of the drivers of glioblastoma multiforme progression [40,41]. In addition, chemokines are involved in intrinsic tumor growth and metastasis, which together make the infiltration of immune cells in TME an essential factor in the GBM prognosis [16,42]. The dependence of GBM on chemokine signaling is further supported by the results of profiling the expression of chemokine receptors in gliomas, which showed that their diversity and mRNA expression levels increase with the glioma grade [43]. Therefore, further development of cell therapies, such as CAR-T cell therapy for the treatment of GBM patients [44,45], depends on developing effective ways to alleviate the immunoselective and/or immunosuppressive capabilities of the tumor microenvironment. As for the glioblastoma multiforme, the TME is cellularly diversified. It consists of normal and reactive astrocytes, pericytes, fibroblasts, myeloid cells, glioma-associated microglia/macrophages, and nontransformed neural stem cells, in addition to glioma tumor cells and glioblastoma stem cells (GSCs) [46]. Moreover, within the perivascular niches of capillaries or arterioles, the glioblastoma TME is also composed of endothelial cells, which are in direct contact with GSCs [47]. The results of our study clearly indicate that it is the presence of a high diversity of TME cells in GBM that explains the broad chemokine expression profile obtained in the analyses. Using bioinformatics tools, we examined chemokine expression in more than 800 tumors from the TCGA and CGCA cohorts, as well as from the study by Gozdz et al. [25], yielding a rich and diverse profile that was highly consistent across all datasets analyzed, with CXCL16, CX3CL1, CXCL14, CCL2, and RARRES2 in the top five expressed chemokines. Overall, the expression of these chemokines is associated with processes promoting GBM progression, including angiogenesis, chemotaxis of MDSCs and TAMs, polarization of macrophages to M2 phenotype and Th2 cells to Tregs, as well as the migration and invasion of glioblastoma cells and GSCs (summarized in [14]). Additionally, it was demonstrated that chemerin (a protein product of RARRES2 expression) promotes mesenchymal features of GBM [48]. Furthermore, re-analysis of single-cell RNA-Seq performed on the basis of clustering by cell signatures [34,35] allowed us to demonstrate that the vast majority of TME cells contribute to the formation of the GBM chemokine microenvironment. Although it is obvious that the profile of chemokines expressed by immune cells was quite rich, GBM cells expressed CX3CL1, RARRES2, and CXCL14 in high amounts, while high expression of CCL2 was detected in GBM, myeloid cells, and pericytes. Interestingly, all of these chemokines secreted by cancer cells are known to promote not only the chemoattraction of immune cells and their polarization to a pro-tumorigenic phenotype, but, as demonstrated previously, they also act through an autocrine pathway, regulating the functions of the GBM cells from which they are secreted [48,49,50]. While those of the chemokines identified in the UMAP analysis as expressed by tumor cells were similarly expressed in the transcriptome of spherical ex vivo cultures (with the exception of the CXCL14 decrease), our study showed that primary cultures dramatically lose the abundance of the chemokine profile of the tumor tissue from which they are derived. The decrease in CXCL14 expression in primary cultures may have been due to several factors, e.g., hyperoxic culture conditions compared to tumor tissues (as CXCL14 is reported to be overexpressed in hypoxia [51]), the fact that its expression is regulated by GBM TME cells [50], and the fact that in tumors, it is additionally highly expressed by cancer-associated fibroblasts [50], a cell type not included in our analysis. Nevertheless, the decrease in expression of other chemokines in primary GBM cultures was most likely related to the loss of diversity of TME cells and their interaction with glioblastoma cells. Therefore, our research has indicated that for the study of new cellular therapies, as well as to explore the details of GBM immunobiology, it is critical to correctly reproduce its chemotactic landscape in vitro, using technology that enables us to reflect the cellular and structural diversity of the TME, which is provided by 3D bioprinting.

Evidence for this was provided by our reanalysis of the Tang et al. (2020) data [3], in which we demonstrated that the use of primary 3D-bioprinted GBM cells along with several TME cell types largely reproduced the multiplicity of expressed chemokines. However, based on the differential gene expression analysis, it can be concluded that a greater effect on chemokine expression was observed with the use of 3D-bioprinting technology on myeloid cells rather than on GBM. Notably, spherical cultures of GBM cells were found to express higher levels of CXCL14, CXCL12, and CX3CL1 compared to those of cultures of the same cells in both 3D-bioprinting systems (TRI and TETRA). The use of macrophages differentiated from iPSCs in the 3D-bioprinting system restored CXCL12 expression to the level of spherical cultures but had no effect on either CXCL14 nor CX3CL1 expression. It is therefore highly possible that high expression of these two chemokines is a feature of glioblastoma stem cells, as other studies have also suggested [50,52]. Moreover, in view of the fact that the expression of membrane-bound CX3CL1 and its receptor mainly involves outer layer cells of spherical cultures (suggesting its involvement in adhesion processes as well) [52], it is possible that placing these cells in structures with forced architecture, a different extracellular matrix, as well as interactions with TME cells make them lose the original stem cell-like features, but this requires further studies. 

Nevertheless, based on the large number of chemokines expressed by the pericytes of GBM tumors, and in view of the results of the analysis from 3D-bioprinted systems, we performed experiments in a simplified 3D-bioprinting model, in which we focused on the interactions of pericyte precursors, i.e., MSCs, and two GBM cell lines (U-251 and DK-MG) and examined the levels of chemokines secreted by them in relation to the biomaterial used and culture time. We aimed to evaluate the chemokine secretion level also in hypoxia, since hypoxia is one of the main features and determinants of the aggressive phenotype of GBM [53]. As the microenvironment of GBM tumors is heavily hypoxic (due to the rapid proliferation and expansion of the tumor mass, which the vascular system, despite growth stimulation, is unable to reach or ceases to reach due to the production of non-functional new vessels prone to thrombosis and occlusion), the lack of oxygen consequently leads to the occurrence of foci of massive cell death by necrosis, observed histologically and being one of the identifying features of GBM [54]. Tumor cells thus escape from severely hypoxic areas, directing toward those richer in oxygen near functioning blood vessels. There they form dense fronts of elongated cells called pseudopalisades, arranged in rows at the periphery of necrotic areas. In our study, we used culture in hypoxia with 1% oxygen, which corresponds to that between the necrotic and pseudopalisade areas of the GBM tumors [53]. However, due to the fact that oxygen levels in a medium of even a simple monolayer cell culture are slightly lower than in the air in which they are maintained [55], it should be considered that the 1% O_2_ we choose may actually be more representative of severe hypoxia or even necrotic areas of the GBM. Our results of chemokine secretion analyses clearly demonstrated that the secretion of most chemokines by cells of both GBMs and MSCs cultured separately in 1% O_2_ significantly decreases compared to those cultured in 21% O_2_, but for the vast majority of them, in the 3D-bioprinted co-culture systems, it is preserved at least to some extent. Given that in our 3D-bioprinted system, GBM cells and MSCs were not in direct contact with each other, it is most likely that paracrine signals are responsible for sustaining chemokine secretion in GBM microenvironment cells under very limited oxygen availability, which only confirms their high mutual dependence and functional connection. Probably due to the use of highly hypoxic conditions, in our analyses, even the levels of chemokines with well-established overproduction in hypoxia (such as CXCL8) still did not exceed those at 21% O_2_, and it is certainly worth noting that they still remained very high in the co-cultures for CXCL8, CCL2, CX3CL1, chemerin, and CXCLIt is also interesting that in the 3D-bioprinted GBM cell lines, we obtained dramatically higher levels of secreted CXCL8 and, to a lesser extent, also CCL3, CCL4, chemerin, and CXCL9 than we determined in neurosphere primary cultures (Supplementary Figure S2). Conversely, the secretion of CXCL12, CXCL16, and CCL5 was significantly lower in all hypoxic culture conditions. In our view, the decrease in CXCL12 secretion by GBM cells under such low oxygen conditions, as well as the increased expression of CXCR4 (its receptor) in response to hypoxia [56], may play a functional role in forcing tumor cells to migrate along the CXCL12 gradient, directing them to perivascular areas where oxygen conditions are improved. It is possible that this mechanism also functions in the migration of GBM cells along the CXCL16–CXCR6 axis, as has already been described, for example, for breast cancer MDA-MB-231 cells [57]. 

Finally, our study also indicates how much the choice of biomaterial used and the cell line affects the results of chemokine testing in 3D-bioprinting systems. It may be surprising how much the two GBM lines differ in their chemokine secretion levels. In our experimental setup, the U251 line secreted higher amounts than DK-MG of all chemokines tested except CXCLIt is very interesting that U251 cultured alone hardly secreted CXCL12, while DK-MG secreted its substantial amounts, but when co-cultured with MSCs, U251 with MSCs secreted higher amounts than DK-MG with MSCs. In addition, we noted a large effect of biomaterial type on the secretion of multiple chemokines in favor of CELLINK Bioink, but this effect was much more pronounced in case of MSCs than in GBM cells, and it appears that in the case of co-cultures, this was also an MSC-related effect. 

This study has some limitations. It is important to note that the 3D-bioprinting system we employed is very simplistic, involving the use of only two of several cell types from the GBM tumor microenvironment. While this is a convenient way to study the interactions between these two cell types, it must be acknowledged that in the organism, these interactions will be further shaped by many other factors. Therefore, our results can only partially relate to chemokine secretion within the tumor. In addition, the use of GBM cell lines instead of primary cells, while allowing for greater reproducibility within the study and between research centers, will never fully reproduce all aspects of tumor cell biology. In addition, when using primary MSCs from a single donor, it must be recognized that these cells exhibit a relatively high interindividual variability. Therefore, the degree and extent of their response to interactions with GBM cells in other donors may actually vary. There is a great need to further improve in vitro models, including 3D bioprinting, and validate these results in other models based on primary GBM cells from patients. Considering these and other limitations associated with the use of in vitro models, we would like our results from 3D bioprinting to be considered in the context of bioinformatic analysis of chemokine expression profiling in GBM tissues. Currently, this is one of the prime research tools for characterizing, at least to some extent, the complex and dynamic system that a tumor actually is.

## 5. Conclusions

In conclusion, this report lays the groundwork for future investigations into the mechanisms shaping the chemokine microenvironment associated with glioblastoma multiforme pathobiology and its targeting in novel immunotherapies and/or other types of precision medicine targeting these tumors. We provide new insights into the impact of mixed cell cultures, with special regard to perivascular mesenchymal cells, but also the technological aspects of the bioprinting process on the chemotactic secretome in the studied model. Based on our observations, further development of multicellular cultures or adaptation of currently existing 3D-bioprinted GBM-TME models with appropriate bioinformatics analysis of the data is warranted.

## Data Availability

The raw data supporting the conclusions of this article will be made available by the authors on request.

## Supplementary Materials

The following supporting information can be downloaded at: https://www.mdpi.com/2073-4409/13/17/1404/s1, Figure S1. A continuation of Figure 5 from the article. Figure S2. Secretion of selected chemokines by ex-vivo spherical cultures of primary glioblastoma multiforme (GBM) cells; Table S1: Detailed results of statistical analysis of chemokine secretion from Figure 7 obtained using the mixed-effects model with Geisser-Greenhouse correction in GraphPad Prism software (version 10.3.0).
