# Supplementary material for: 3D-Bioprinted Co-Cultures of Glioblastoma Multiforme and Mesenchymal Stromal Cells Indicate a Role for Perivascular Niche Cells in Shaping Glioma Chemokine Microenvironment
Source: Cells. 2024 Aug 23;13(17):1404. doi: 10.3390/cells13171404 (PMC11393941; doi:10.3390/cells13171404)
Supplement: Supplementary file 1 [file cells-13-01404-s001.zip › Supplementary Figures.pdf]

Supplementary Materials

3D-Bioprinted Co-Cultures of Glioblastoma Multiforme and Mesenchymal Stromal Cells Indicate a Role for Perivascular Niche Cells in Shaping Glioma Chemokine Microenvironment

Katarzyna Zielniok, Kinga Rusinek, Anna Słysz, Mieszko Lachota, Ewa Bączyńska, Natalia Wiewiórska-Krata, Anna Szpakowska, Martyna Ciepielak, Bartosz Foroncewicz, Krzysztof Mucha, Radosław Zagożdżon and Zygmunt Pojda

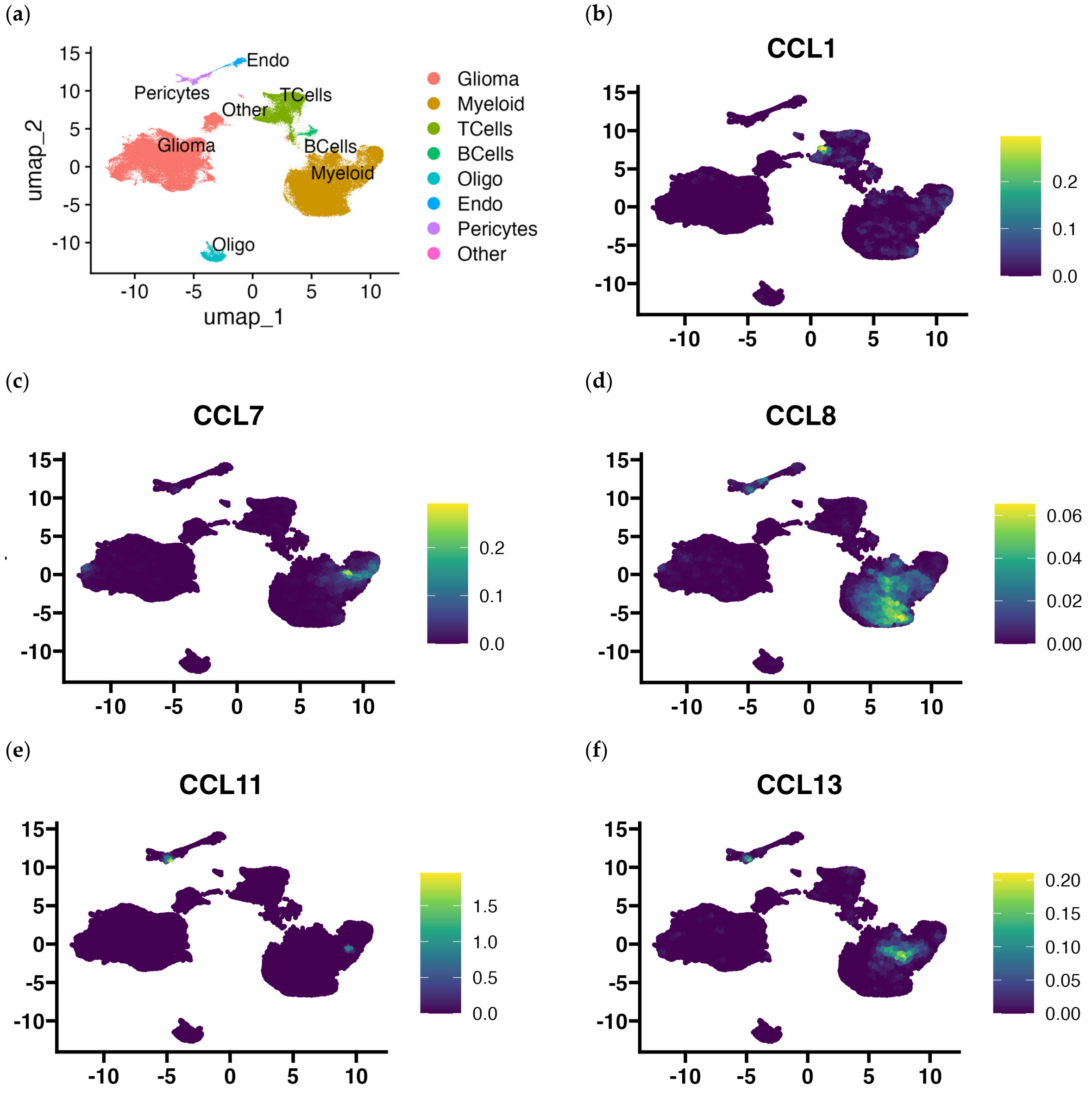

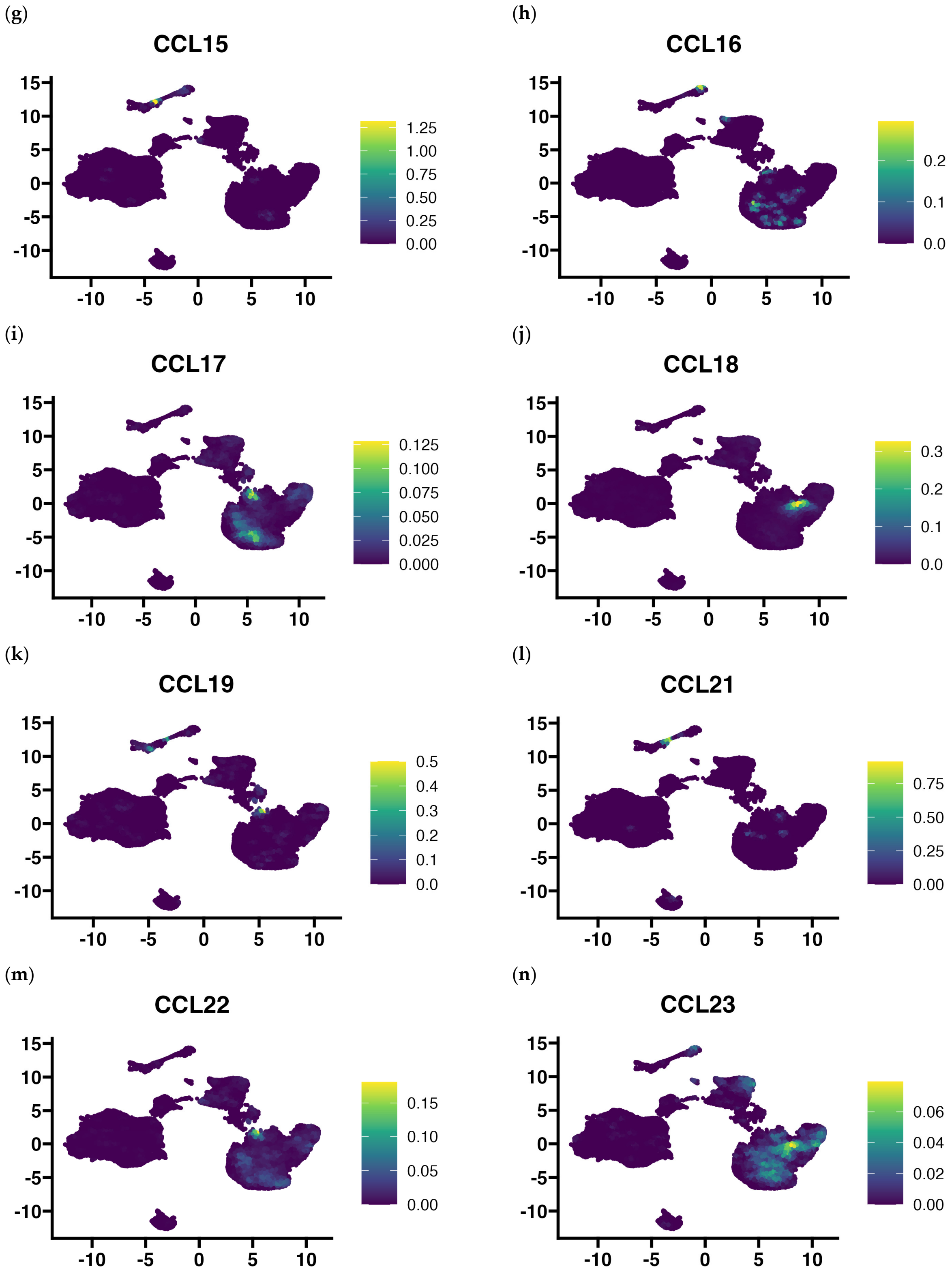

(g)
CCL15
1.25
1.00
0.75
0.50
0.25
0.00
(h)
CCL16
0.2
0.1
0.0
(i)
CCL17
0.125
0.100
0.075
0.050
0.025
0.000
(j)
CCL18
0.3
0.2
0.1
0.0
(k)
CCL19
0.5
0.4
0.3
0.2
0.1
0.0
(l)
CCL21
0.75
0.50
0.25
0.00
(m)
CCL22
0.15
0.10
0.05
0.00
(n)
CCL23
0.06
0.04
0.02
0.00
15
10
5
0
-5
-10
-10
-5
0
5
10

(o)

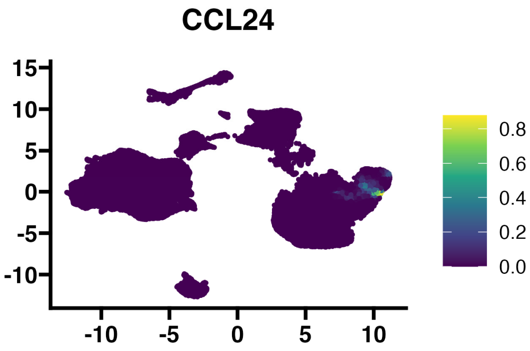
CCL24
15
10
5
0
-5
-10
-10 -5 0 5 10
0.8
0.6
0.4
0.2
0.0

(p)

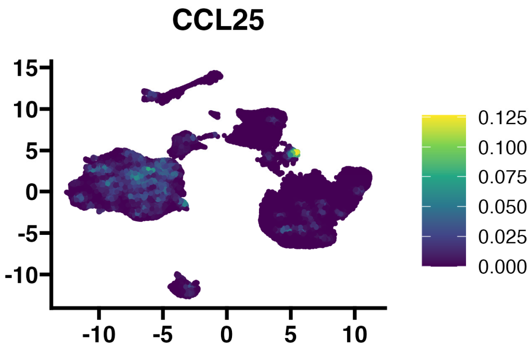
CCL25
15
10
5
0
-5
-10
-10 -5 0 5 10
0.125
0.100
0.075
0.050
0.025
0.000

(q)

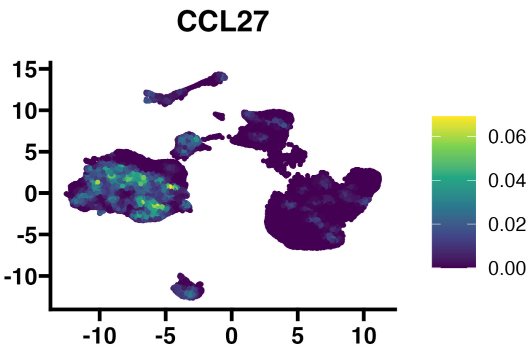
CCL27
15
10
5
0
-5
-10
-10 -5 0 5 10
0.06
0.04
0.02
0.00

(r)

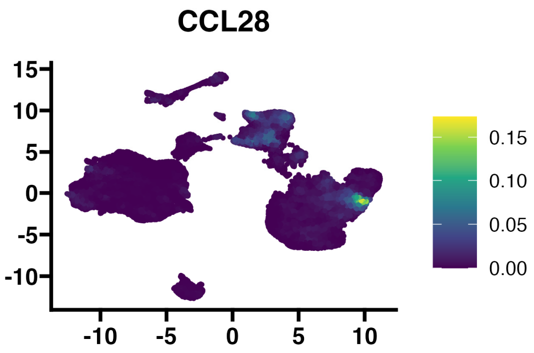
CCL28
15
10
5
0
-5
-10
-10 -5 0 5 10
0.15
0.10
0.05
0.00

(s)

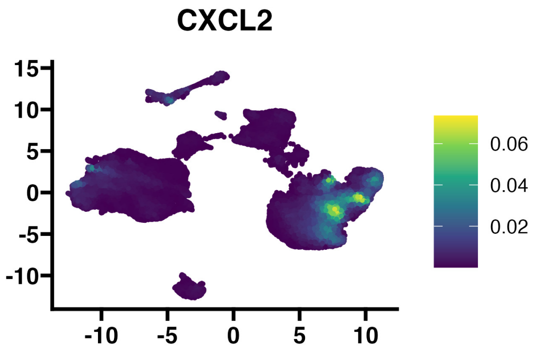
CXCL2
15
10
5
0
-5
-10
-10 -5 0 5 10
0.06
0.04
0.02

(t)

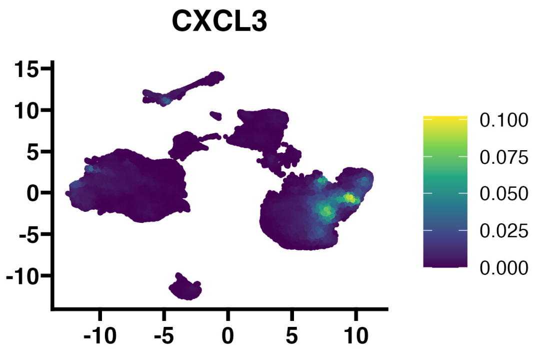
CXCL3
15
10
5
0
-5
-10
-10 -5 0 5 10
0.100
0.075
0.050
0.025
0.000

(u)

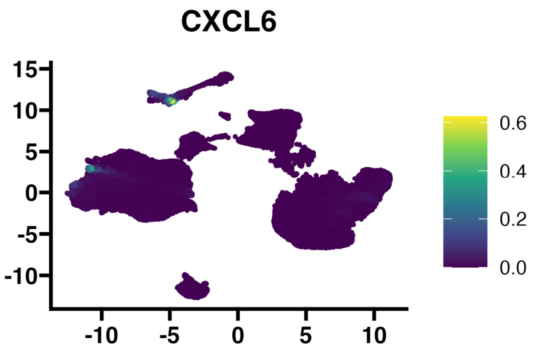
CXCL6
15
10
5
0
-5
-10
-10 -5 0 5 10
0.6
0.4
0.2
0.0

(v)

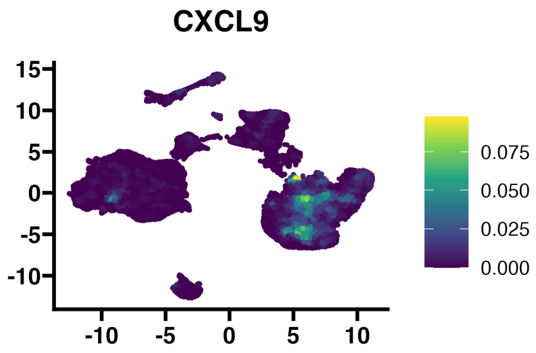
CXCL9
15
10
5
0
-5
-10
-10 -5 0 5 10
0.075
0.050
0.025
0.000

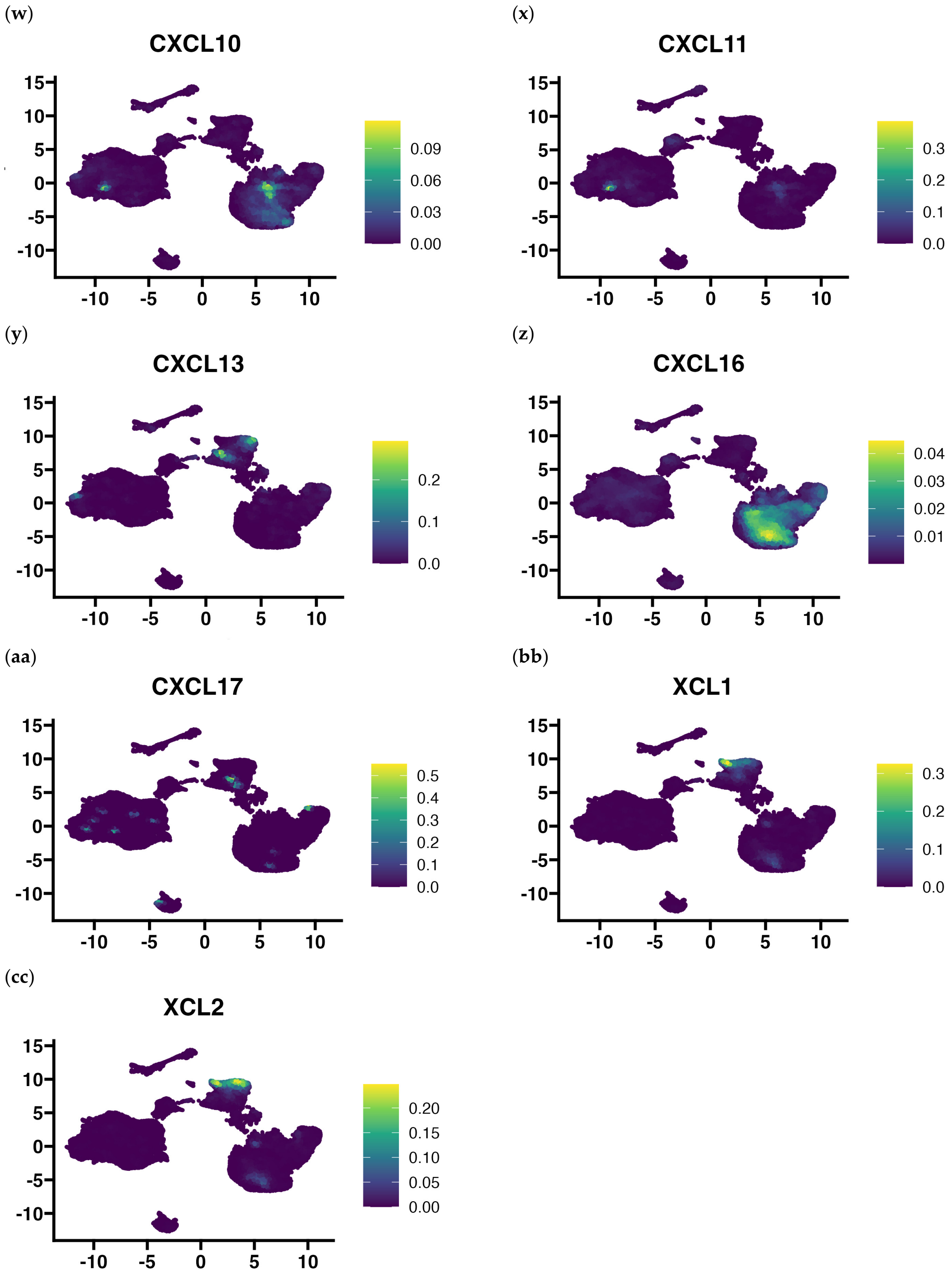

Suppementary Figure S1. A continuation of Figure 5 from the article. The expression plots of remaining chemokines that were not included within the main manuscript, but are derived from the same analysis. Chemokine expression at a single-cell level from 2 low grade glioma and 16 primary glioblastoma multiforme tumors (201 986 cells) projected onto the tSNE (t-distributed stochastic neighbor embedding) plots, annotated according to the cell signature clustering data from the original article by Abdelfattah et al.

[26, 27] as show in (**a**) into: glioma cells, myeloid cells, T lymphocytes, B lymphocytes, oligodendrocytes, endocytes and pericytes, and others. Single cell RNA-Seq data was downloaded from Single Cell Portal (https://singlecell.broadinstitute.org/single_cell), accession date 13th March 2024 (SPC1985/GSE182109) [25] and imported into Seurat (v4.9.9.905). Visualization was performed using Nebulosa (v1.10.0) [28].

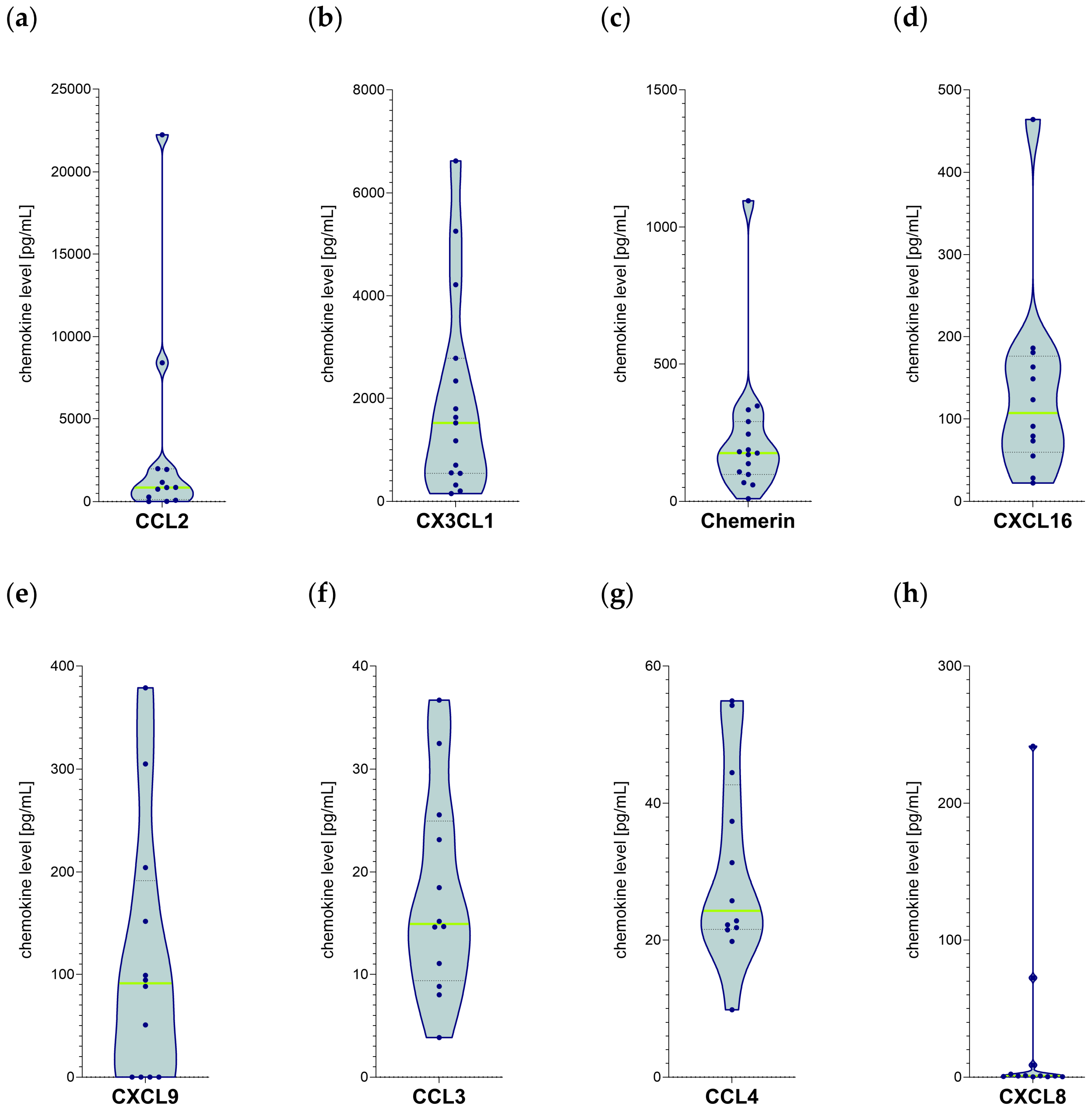

Supplementary Figure S2. Secretion of selected chemokines by *ex-vivo* spherical cultures of primary glioblastoma multiforme (GBM) cells. Cells of 12 donors of GBM were kindly provided by Ilona Kalaszczyńska, Agata Góźdź and Patrycja Szpak. Cultures were derived from GBM tumors during surgical resection as described in [14]. Neurospheres were cultured for 48h in 5 ml of neurobasal medium (DMEM/F-12 GlutaMAX + 2% B-27 supplement + 20ng/mL bFGF + 20ng/mL EGF + 0.5% antibiotic-antimycotic solution, all ThermoScientific) in 25 cm^3 bottles in non-adherent spherical cultures (Sarsteadt). The medium was then collected, centrifuged (4500 × rpm, 4 minutes) and frozen at -80°C for luminex analysis. Chemokine secretion profiling was performed using the "R&D 17-plex Human Luminex Discovery Assay" kit, and results were normalised by chemokine levels in the neurobasal medium. Secretion results

of (a) CCL2, (b) CX3CL1, (c) chemerin, (d) CXCL16, (e) CXCL9, (f) CCL3, (g) CCL4 and (h) CXCL8 are presented in [pg/mL] and calculated based on the number of cells from each culture as secretion per 1 million cells. Violin plots were marked with dots of each individual result and the green line indicates the median.
